# The omics revolution: beyond genomics. A meeting report

**DOI:** 10.1186/s12014-020-9266-9

**Published:** 2020-01-24

**Authors:** E. C. Nice

**Affiliations:** Department of Biochemistry and Molecular Biology, Monash University, Clayton, VIC 3800 Australia

**Keywords:** Biosensors, CryoEM, HUPO, IP and legal issues, Mass spectrometry, Proteomics, Proteogenomics, Personalised/precision medicine

## Abstract

The “omics revolution: beyond genomics” satellite meeting, run under the auspices of the Australian Peptide Association, The Human Proteome Organisation (HUPO) and the HUPO Australia/New Zealand Chromosome 7 initiative, was held at the Oaks Resort, Port Douglas, Queensland, Australia, on 8th September 2019, immediately prior to the 13th Australian Peptide Conference. The meeting, which had over 100 participants representing Australasia, Europe and America, focused on recent advances in omics-related technologies, including mass spectrometry, biosensors and CryoEM, which will assist in the clinical translation of proteomics towards precision/personalized medicine. An overview of the conference and a summary of the oral presentations are presented.

## Introduction

This one day satellite meeting entitled “The omics revolution: beyond genomics” was held at the Oaks Resort, Port Douglas, Queensland, Australia, on 8th September 2019, immediately prior to the 13th Australian Peptide Conference. The meeting was run under the auspices of the Australian Peptide Association, The Human Proteome Organisation (HUPO) and the HUPO Australia/New Zealand Chromosome 7 initiative. It followed on from the previous successful meetings in this series that were held in conjunction with the 2013 Australian Peptide Conference in Penang, Malaysia (Proteomics Forum, Malaysia), the 2015 meeting in Kingscliff, Australia (The “Omics” Revolution: Uncovering Proteome Complexity), and the 2017 meeting in Noosa, Australia (The Omics Revolution: Towards Personalised Medicine). The program committee comprised Prof Ed Nice (Monash University, Melbourne, Australia), Assoc. Prof Vera Ignjatovic (Murdoch Childrens Research Institute, Melbourne, Australia), Assoc. Prof Michelle Hill (QIMR Berghofer Medical Research Institute, Brisbane, Australia) and Dr Christie Hunter (SCIEX, Foster City, USA). The aim of the meeting was to inform the audience on recent advances in the role of proteomics–based technologies for clinical translation while also identifying possible hurdles for their uptake. Speakers were chosen based on recent exciting output from their laboratories. This year there were over 100 delegates from 16 countries with almost half coming from overseas. This included strong representation from China (16), Japan (7), USA (6), and Singapore (5). Delegates represented academia, biotechnology, the pharmaceutical industry and the legal and patent fraternity. In line with the current move towards a systems biology approach [1] and clinical translation [2], the meeting focused on the key role proteomics and other omics-related technologies will play in the role out of personalised/precision medicine. There were 14 oral presentations spread across 4 sessions that are summarized below.

## Session A. Opportunities and hurdles in proteogenomics

The opening session commenced with a presentation on the role of long non-coding RNAs (lncRNAs) in head and neck squamous carcinoma (HNSCC) entitled “The oncogenic lncRNA RP11-499F3.2 promotes tumorigenesis and cetuximab resistance via activation of Wnt2B signalling” from the group of Prof Hanmei Xu at China Pharmaceutical University, Nanjing, China. lnc-RP11-499F3.2 is associated with poor clinical survival, tumour proliferation and metastasis in HNSCC. They showed that RP11-499F3.2 was overexpressed in cetuximab-resistance patient-derived xenograft (PDX) tumours that progressed following cetuximab treatment. They proposed that RP11-499F3.2 functions as a competing endogenous RNA (ceRNA) for miR-6817, thereby maintaining Wnt2B stability and promoting downstream targets of the Wnt/β-catenin pathway.

This was followed with a talk by Dr Atsuhiko Toyama from Shimadzu Corporation, Singapore on “Mediator Lipidomics: Towards Comprehensive Metabolic Profiling of Eicosanoids and Related Fatty Acids”. Using their UHPLC-MS/MS platform, they presented a targeted method for comprehensive metabolic profiling of eicosanoids and related fatty acids that mediate autocrine, paracrine and endocrine signalling in diverse pathophysiological systems. A method was developed comprising 326 MRM transitions, using a 20 min chromatographic separation, enabling the measurement of 196 fatty acid metabolites. Eighteen deuterium-labelled analogues were used as internal standards. This method was applied to a mouse model of inflammatory response. Multivariate analysis of around 100 targets detected from mice serum samples yielded characteristic disease-related profiles.

Major hurdles facing many researchers are those of funding, patent protection and regulatory requirements. These topics were addressed from a legal perspective in the next two talks. Dr Jane Nielson (University of Tasmania, Hobart) presented on “Reforming the Regulatory Environment for Innovative Health Technologies: Identifying Congestion and Filling Gaps”. Her hypothesis was that previously unforeseeable advances in health innovation are testing the capacity of regulators to ensure patient safety while those at the research coalface are faced with myriad regulatory hurdles exerting significant stress on efficiently designing and conducting biomedical research. She pointed out that current regulatory frameworks assume a linear model of innovation that is far removed from current practice. She used an ARC-funded Discovery project as a model. This aimed at mapping the current regulatory environment to identify gaps, areas of regulatory congestion, or over-regulation by interviewing key stakeholders. Three technologies (genome editing, biologics and 3D bioprinting) were taken as exemplars. They were selected because, taken together, they encounter the gamut of regulatory issues facing both health innovators and regulators.

The final talk in this session on “Patentomics: personalised protection” (jokingly called “Condomics” by one of the session chairs) was given by Dr Simone Vink, a patent attorney with Davies Collison Cave (Davies Collison Cave Pty Ltd, Milton, QLD, Australia), who has a background in bioactive peptides in animal venoms and the synthesis and functional characterisation of orally stable peptide therapeutics. She pointed out that the importance of obtaining patent protection for products associated with precision/personalised medicine is increasing with the rise in the development and use of such inventions. Importantly patent specifications and filing strategies must be tailored for each specific application to obtain optimal protection. However, some recent court decisions have confounded the protection of diagnostics and natural products, with key differences between Australian, European and American patent law. Using a number of examples of how these recent decisions have impacted on patent applications for diagnostics and natural products such as peptides and proteins (e.g. the case of the Association for Molecular Pathology v. Myriad Genetics, Inc.), she demonstrated how the use of diverse and non-traditional approaches (e.g. manipulation of DNA sequences) could be used to overcome various rulings.

## Session B. Proteomics: towards the clinic

This session focussed on a number of disease related applications. Dr Larissa Lago, from the group of A.Prof Blaine Roberts at the Florey Institute of Neuroscience and Mental Health, Melbourne, Australia, led off the session with a presentation on “When metalloproteins go rogue: application of metalloproteomics to understand neurodegeneration”. This group has focussed on the development of tools for the direct identification and quantitation of individual metalloproteins in biological samples. Using metalloproteomics, surprising insights into the role of metalloproteins in amyotrophic lateral sclerosis (ALS) and Alzheimer’s disease have been obtained, leading to the first in-class clinical trial for ALS and Parkinson’s disease.

This was followed by a presentation entitled “ProCan: from cancer tissue proteomics to clinical application” by Dr Rosemary Balleine, a pathologist working with ProCan^®^ at The Children’s Medical Research Institute, The University of Sydney, Westmead, Australia. ProCan is positioned to undertake a large-scale survey of the cancer tissue proteome over a 5–7 year timeframe. With access to 70,000 tissue sections covering a wide range of cancer types, the overall goal is to assemble a comprehensive proteomics knowledge-base that, in combination with clinical information and other ‘omics” data, will support both discovery and translational aspects of the development of biomarkers for clinical use. Dr Balleine presented an overview of the facility and the development of the techniques used at ProCan. In what has been called “industrial scale proteomics” ProCan uses novel pressure cycling technology (PCT) to optimise tissue sample preparation (both formalin-fixed paraffin-embedded (“FFPE”) and fresh frozen biopsy tissue samples) coupled with data independent analysis (DIA) using SWATH (sequential window acquisition of all theoretical mass spectra) technology for deep interrogation of individual proteomes. Data can be aligned with the corresponding immunohistochemistry (IHC) and de-identified clinical data and analysed with the help of advanced bioinformatics. Large amounts of data are being generated, both from the mass spectrometry and digital pathology images. For each MS analysis, around 1 gigabyte (GB) of data is generated. By the end of the program, it is estimated that ProCan will have more than 200 terabytes (TB) of data just from MS. Handling of the “Big Data” that will be generated to support precision medicine remains a major challenge. ProCan is part of the Cancer Moonshot program that was established by the Obama administration in the US in 2016 with the goal of significantly accelerating cancer research through international collaboration and data sharing to make more therapies available to more patients, while also improving our ability to prevent cancer by detecting it at an early stage when treatment is more successful. There are currently 13 countries and 33 institutions involved.

A.Prof. Michelle Hill of the QIMR Berghofer Medical Research Institute, Brisbane, QLD, Australia, presented her recent data on “Towards liquid biopsy screening for esophageal adenocarcinoma (EAC)”. While EAC is a relatively rare cancer (about 1500 deaths per annum in Australia), the incidence is markedly increasing. EAC is thought to develop from asymptomatic Barrett’s esophagus (BE) with a low annual rate of conversion. Michelle’s studies have focussed on the potential of disease-related glycoproteins as potential markers of early stage disease. To assist in this goal a lectin magnetic bead array (LeMBA) coupled with a mass spectrometry biomarker pipeline was developed which revealed novel potential diagnostic biomarkers for EAC [3]. The data were further validated in independent cohorts [4] and a candidate biomarker panel for BE surveillance developed. These data also revealed the role of Complement Component C9 JAC lectin that was validated by IHC and its role in EAC development investigated.

The final talk in this session on “Alzheimer’s Disease—an Assay Odyssey” was given by Dr Darren Weber from Quest Diagnostics, San Juan Capistrano, CA, USA. Quest Diagnostics is one of the largest clinical diagnostics company worldwide that offers a number of MS-based clinical assays. There is currently an urgent need for better diagnostic tests to detect Alzheimer’s disease at an earlier stage that this group are addressing. Darren overviewed the progression of Alzheimers disease and discussed the road to developing and commercializing a mass spectrometry based Abeta 1–42/1–40 ratio cerebral spinal fluid (CSF) assay using SRM. Current ELISAs have very large variance between them making cross lab comparisons difficult. The use of mass spectrometry should improve cross lab comparisons. He also described the subsequent development of an Abeta 1–42/1–40 ratio plasma-based screen using Quanterix’s SIMOA technology [5]. Both tests are now being used in patient care and to assist clinical trial enrolment.

## Session C. Antibody and biosensor-based applications for proteomics

Antibody and biosensor based applications are another important component of the proteomics toolbox, antibodies being one of the four working pillars of HUPO (MS, knowledge base, antibodies and pathology). Prof Malcolm Buckle (CNRS, Cachan, Ile de France, France) led off the session with a talk entitled “Examples of how a novel surface chemistry enables gold surface biosensors to carry out selective early diagnosis: In search of Eldorado”. This talk addressed the important topic of reducing non-specific binding on biosensor surfaces in complex biological samples, allowing analytes to interact specifically and exclusively with their target molecule. He detailed the use of a proprietary chemistry called general liquid interface specific surfaces (GLISS), using surface plasmon resonance (SPR) to demonstrate the efficiency of GLISS treated surfaces. GLISS demonstrably overcame non-specific interactions with the sensor surface and allowed bio-specific detection in complex media such as blood and cell and bacterial extracts. Importantly GLISS allowed the detection of analytes in such complex media at the limit of detection of the biosensor, with non-specific interactions reduced by up to 200-fold. Examples presented included DNA/DNA hybridization, antibody-antigen interactions and real time analysis of bacterial growth under antibiotic selection conditions.

This was followed with a presentation by Prof Mibel Aguilar (Monash University, Clayton, Australia) on “Optical biosensors that provide new insights into biomembrane-mediated events”. This presentation highlighted the use of optical biosensors together with other biophysical techniques to analyse biomembrane interactions. It demonstrated how this new understanding could be a platform for both lipidomic analysis and high-resolution imaging of biomembrane structure and function and could show new insights into antibiotic resistance. Data using two different readouts [SPR and dual polarisation interferometry (DPI)] were presented. Importantly the data identified factors that drive the structural changes in both the membrane and the peptide, measure and define the critical concentration required for membrane lysis, track the changes in membrane structure disorder as a function of the mass of bound peptide in real time and define a multi-state model of membrane disruption.

The final talk in this session was given by Dr Cecilia Lindskog (Department of Immunology, Genetics and Pathology, Human Protein Atlas, Uppsala University, Uppsala, Sweden) who outlined the current status of the Human Protein Database. This database is based on integration of transcriptomics and antibody-based proteomics and constitutes the largest and most comprehensive knowledge resource for spatial localization of proteins in organs, tissues, cells and organelles. It contributes to the HUPO human antibody initiative. The current release contains data based on 26,371 well-characterised antibodies targeting 17,058 unique proteins (86% of the human proteome based on the current metrics [6]. Currently divided into three different sub-atlases (the Tissue Atlas [7], the Pathology Atlas [8] and the Cell Atlas [9]) it covers a wide spectrum of spatial localization at different levels, as well as addressing the consequence of human genes on patient survival. The database has several potential implications for use in personalized medicine. It constitutes an important starting point for identification of candidate proteins that may have important implications for medicine as it aids in identifying and stratifying high-risk individuals, contributes to further understanding of underlying disease mechanisms and guides treatment modalities. Three new sections have been added to the recent 2019 release: the Blood Atlas, studying proteins in different human blood cell types; the Brain Atlas, describing protein localization in different parts of mammalian brain; and the Metabolic Atlas, focusing on proteins involved in metabolic pathways. The Human Protein Atlas constitutes a comprehensive stand-alone open-access resource available to researchers worldwide. It will clearly help to accelerate efforts in completing the first draft of the human genome and in finding novel biomarkers addressing future needs in personalized healthcare.

## Joint session with the Australian peptide conference. Advancing proteomics technologies

The last session was held jointly with the 13th Australian Peptide Conference and comprised three talks on advancing proteomic technologies. Dr Christie Hunter (SCIEX, Redwood City, CA, USA) discussed “Accelerating data independent analysis (DIA) using fast microflow LC gradients for large scale quantitative proteomics”. Christie has had a long involvement in the development of SWATH DIA technologies. SWATH allows comprehensive detection and quantitation of virtually every detectable compound in a sample. Importantly a complete digital archive is generated which can be repeatedly interrogated, allowing sample re-analysis using alternative settings without having to re-run it on the MS. Christie showed that by using higher flow rates, samples can be loaded faster, and the trap and separation column washed and equilibrated faster with rapid gradient formation, allowing much faster run times to be achieved. Using complex digested cell lysates, SWATH acquisition experiments were performed using gradient times ranging from 5 to 45 min. Protein quantitation results were then assessed. Fast MS/MS acquisition rates were found to be critical because this enabled more, smaller variable Q1 windows to improve S/N for quantitation. Optimized methods were then used to compare the quality of quantitation between long and shortened gradients. Similar fold change values were measured confirming accelerated gradients can be used for industrialized quantitative proteomics.

This was followed by A. Prof. Tara Pukala (School of Physical Sciences, University of Adelaide, Adelaide, SA, Australia) who reported on “Ion mobility-mass spectrometry: An added dimension for structural biology in the context of protein aggregation”. A precise network of interacting biomolecules, including proteins, DNA and lipids, dictate function and control all biological processes. Consequently, to understand these processes and offer potential for intervention, for example in the treatment of human diseases, it is useful to have an understanding of the molecular components and their binding interactions. However, structural characterisation of such complex, heterogeneous and dynamic systems remains a challenge, in part due to analytical limitations of current structural biology approaches. This is particularly true in the context of protein misfolding diseases arising from the failure of a specific peptide or protein to adopt, or remain in, its native functional conformational state. Despite extensive efforts to identify the mechanisms by which such diseases result, the way in which natively soluble proteins misfold to produce toxic aggregates is still not fully understood, and a lack of atomic level understanding has hampered rational design of therapeutics. Prof Pukala explained the principles of ion-mobility mass-spectrometry (IM-MS) and how it has emerged as a complementary tool for biomolecule structure determination, being in some cases a structural biology method in its own right. It is capable of defining identity, stoichiometry, size, structural arrangement and subunit interactions in a biomolecular assembly in a single experiment. She presented examples, primarily focussed on the proteins alpha synuclein and amyloid beta (associated with Parkinson’s and Alzheimer’s disease respectively), showing how mass spectrometry has offered new insights into the understanding of protein aggregation pathways. The application of mass spectrometry to investigate the interaction of these proteins with small molecules and lipids was also highlighted. In summary, these data revealed new insight into the role and design of small molecule amyloid inhibitors, the importance of lipids in the aggregation process, and the ability of protein chaperones to stabilise dynamic disorder for misfolding proteins.

The final talk in the meeting was given by Prof James Whisstock [ARC Centre of Excellence in Advanced Molecular Imaging, Monash University, Clayton, VIC, Australia (Australia’s first automated state-of-the-art Electron Microscopy Unit)] on “New cryo-EM methods present exciting challenges for the ‘Omics communities”. He explained how modern high-resolution Cryo Electron Microscopy (cryo-EM) techniques are providing crucial insights into the structure of macromolecular assemblies both ex situ and within cells (in situ structural biology). Proteins can be observed in native or near-native biologically relevant states, but optimised sample preparation is crucial. While well-behaved protein samples can routinely yield atomic resolution structures in single particle cryo-EM experiments, key challenges arise with dynamic complexes, or with respect to proteins that fail to readily yield suitable cryo-preserved samples. Hybrid approaches, including the application of “omics” technologies to facilitate identification of protein complexes, enable cryo-EM to be used in the analysis of low resolution EM datasets.

## Outcomes

There is a general misconception that genomics is synonymous with personalised/precision medicine. This meeting has clearly demonstrated this is not the case, with examples presented covering a range of important pathologies. It is also important to realise that proteomics is not just mass spectrometry, another common misconception. This was highlighted in a session on biosensor and antibody-based applications. Talks on legal and IP aspects affecting the roll out of precision medicine highlighted another key problem that needs to be addressed.

Proteomics has significantly matured in recent years, with a number of major advances in the field, particularly with respect to throughput, sensitivity, reliability and reproducibility. This will assist in both an improved understanding of disease biology and the development of validated clinical assays to support personalised/precision medicine.

## Data Availability

Not applicable.
